# Association between Severe SARS-CoV-2 Infection and Severe Acute Pancreatitis in Pregnancy and Postpartum

**DOI:** 10.3390/jcm11092554

**Published:** 2022-05-02

**Authors:** Mihaela Mocan, Robert Szabo, Cătălin Constantinescu, Ciprian Cucoreanu, Romeo Ioan Chira

**Affiliations:** 1 Department of Internal Medicine, University of Medicine and Pharmacy “Iuliu Hatieganu”, 400012 Cluj-Napoca, Romania; mihaela.mocan@gmail.com (M.M.); romeochira@yahoo.com (R.I.C.); 2 Department of Internal Medicine, Emergency County Hospital Cluj, Cluj-Napoca 400006, Romania; 3 Department of Anesthesia and Intensive Care, University of Medicine and Pharmacy “Iuliu Hatieganu”, 400012 Cluj-Napoca, Romania; constantinescu.catalin@ymail.com; 4 Department of Anesthesia and Intensive Care, Emergency County Hospital Cluj, 400006 Cluj-Napoca, Romania; 5 Department of General Surgery, University of Medicine and Pharmacy “Iuliu Hatieganu”, 400012 Cluj-Napoca, Romania; cucoreanu_ciprian@yahoo.com; 6 Department of Gastroenterology, Emergency County Hospital Cluj, 400006 Cluj-Napoca, Romania

**Keywords:** COVID-19, pregnancy, acute pancreatitis, retroperitoneal necrosis

## Abstract

Introduction: SARS-CoV-2 infection (COVID-19) affects the respiratory system but is not limited to it. The gastrointestinal symptoms are polymorphic, including diarrhea, vomiting, abdominal pain, and even acute pancreatitis (AP). Pregnant women are more vulnerable to SARS-CoV-2 infection and have a higher risk of severe outcomes of COVID-19. Case report: We present a case report of a 31-year-old primigravid patient at 30 weeks of gestation, unvaccinated, with a medical history of thrombophilia, chronic nephropathy of unknown origin, hypertension, and optic neuropathy with left eye hemianopsia. She was diagnosed with moderate-to-severe COVID-19 and respiratory failure, with indication for cesarean section. Postpartum, she developed severe acute pancreatitis, complicated by peripancreatic and supradiaphragmatic abscesses. After 3 months of hospitalization and eight surgical interventions, the patient was discharged. A short mini-review of the literature is introduced. Conclusion: Pregnant women with cardiovascular comorbidities are prone to severe complications of SARS-CoV-2 infection. Clinicians should be aware of the association of SARS-CoV-2 and AP in pregnant women.

## 1. Introduction

Severe acute respiratory syndrome coronavirus 2 (SARS-CoV-2) infection affects the respiratory system but is not limited to it. This virus affects multiple organs and systems, such as the cardiovascular, hematological, renal, hepatic, and digestive systems. While the respiratory infection manifests with fever, cough, and respiratory failure in critical cases, the gastrointestinal symptoms are polymorphic, including diarrhea, vomiting, abdominal pain, and even acute pancreatitis (AP) [1]. Clinical data suggest that pregnant women are more vulnerable to infection and have a higher risk of severe outcomes of COVID-19 [2,3,4]. Severe acute pancreatitis (SAP) develops in almost 20% of cases of all patients with AP, and it is associated with high mortality (10–85%), even in patients without known comorbidities, due to pancreatic and extrapancreatic necrosis, later complicated by infection and multiple organ failure [5]. According to the revised Atlanta classification system, a positive diagnosis of AP is defined by the presence of any two of the following three criteria: (a) abdominal pain suggestive of acute pancreatitis, (b) a serum lipase level 3 times greater than the upper normal value, and (c) characteristic imaging findings on ultrasound, contrast-enhanced CT scan, or magnetic resonance imaging (MRI) [6]. The most common causes of SAP are biliary calculus and alcohol consumption, but viral infections, such as coxsackie virus, Epstein–Barr virus (EBV), hepatitis A virus, and herpes viruses, are also known to be responsible for SAP [7]. During the last two pandemic years, we have witnessed a resurgence of AP in patients infected with SARS-CoV-2. The emerging literature data suggest that SARS-CoV-2 has an affinity for pancreatic cells, which is mediated by the ACE-2 receptor, located on the membrane of pancreatic cells, and plays the role of viral receptor, allowing the virus to enter the pancreatic cell [8]. The density of ACE-2 receptors in pancreatic cells, in both exocrine cells and islet cells, is even higher than in lung cells, increasing susceptibility to AP and diabetes mellitus in patients with COVID-19 [9]. Wang et al. reported a 17% incidence of pancreatic injury in patients with COVID-19, diagnosed by mildly elevated serum biomarkers (amylase and lipase) [10]. However, Liu et al. found that 7.64% of patients with COVID-19 presented radiological evidence of pancreatic injury on CT scans [9]. An imaging evaluation is not compulsory for the initial diagnosis if the other two criteria are met, but it could be useful to identify the etiology (cholelithiasis or pancreatic cancer, for example) and, later on, to characterize the complications of SAP [11].

## 2. Case Report

### 2.1. Clinical Presentation

We present the case of a 31-year-old primigravid patient at 30 weeks of gestation with a medical history of thrombophilia, chronic nephropathy of unknown origin with marked proteinuria, hypertension, and optic neuropathy with left eye hemianopsia. She was not vaccinated, as she was afraid of the supposed long-term negative effects of the vaccine. The patient developed symptoms of dry cough and shortness of breath 7 days prior to presentation and tested positive for SARS-CoV-2, as confirmed by real-time reverse transcription PCR of a nasopharyngeal swab. The patient did not have any gastrointestinal symptoms at the time of the initial evaluation.

**Figure 1:**
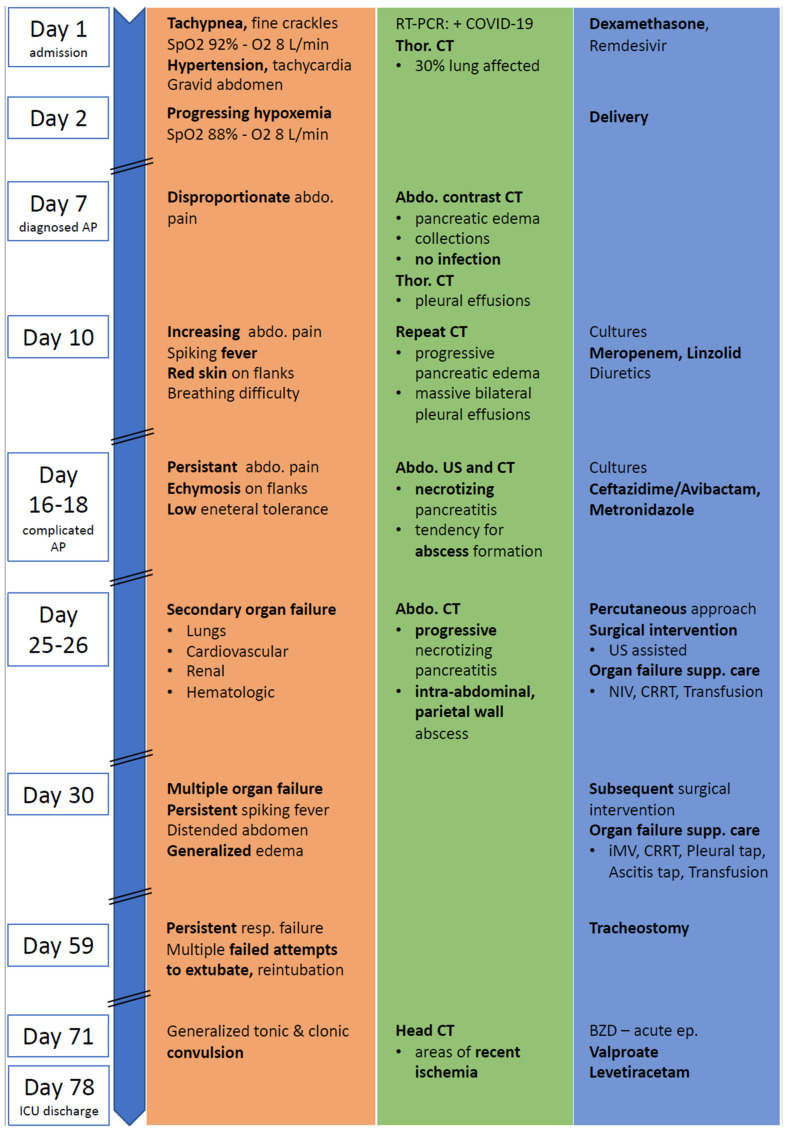
Event timeline from admission to hospital to discharge from intensive care. CT: computer tomography; Abdo: abdominal; Thor: thoracic; AP: acute pancreatitis; US: ultrasound; NIV: noninvasive ventilation; iMV: invasive mechanical ventilation; CRRT: continuous renal replacement therapy; BZD: benzodiazepines; orange represents clinical presentation; green represents lab and imaging investigations; blue represents therapy.

On admission, a physical examination revealed a blood pressure (BP) value of 180/80 mmHg, tachycardia of 110–120 beats per minute (bpm), respiratory rate of 18/min, and hypoxemia defined by a low peripheral saturation in oxygen (SpO2) of 85% in room air and 92% with supplemental oxygen of 8 L/min via a full-face mask. A thoracic auscultation revealed fine crackles in the lower pulmonary fields, bilaterally. Her abdomen was gravid, soft, and nontender. During the 24 h following admission, the health of the patient deteriorated with worsening respiratory failure, SpO2 of 88% with 8 L/min oxygen via full-face mask. The caring obstetrics and gynecology (OG) team decided to perform a cesarean section to deliver the baby. The newborn was male with a birthweight of 1240 g and a 5 min Apgar score of 10. He was isolated from his mother immediately after birth and received formula feeding. As a premature baby with a low birth weight, he was hospitalized in Neonatology ICU for two months and treated accordingly. He was tested negative for COVID-19 using a throat swab specimen with RT-PCR 72 h after birth. An overview and a timeline of the patient’s course of events can be seen in Figure 1.

Following delivery, the patient developed disproportionate abdominal pain with worsening intensity and, therefore, was transferred from the OG intensive care unit (ICU) to the general ICU for patients with COVID-19. The patient was alert, oriented, and hemodynamically stable with moderate abdominal pain. Based on clinical findings, lab and imaging investigations, the diagnosis of acute pancreatitis was established. On day 10, she complained of increasing abdominal pain. She developed spiking fever and breathing difficulty, which coincided with the appearance of red skin on the flanks. As the disease progressed, on day 16, her respiratory and hemodynamic status deteriorated. She developed tachypnoea, tachycardia, and the tendency to hypotension. Furthermore, on day 18, persistent abdominal pain and erythema with areas of ecchymosis on the left flank region were accompanied by a decrease in tolerance for enteral nutrition. Based on these findings, complicated severe acute pancreatitis was suspected. At this stage, a conservative medical approach was adopted.

On day 25, despite supportive and specific treatment, she developed secondary organ failure. Signs of respiratory failure with increasing oxygen demand were accompanied by a decrease in BP to values lower than 20% of her baseline. Deteriorated renal function was indicated by a decrease in urine output (UO). A shift from conservative to invasive treatment was required. Despite adopting a surgical approach, the patient continued to clinically deteriorate. On day 30, her UO decreased to levels below 0,5 mL per body weight per hour (mL/kg/h) and was accompanied by generalized edema, a distended abdomen, and worsening respiratory failure. Dyspnea with increased work of breathing and low oxygen saturations were present and, while on auscultation, low bilateral diminished breath sounds were heard. Spikes in temperature were repeatedly recorded, which indicated the need for subsequent invasive interventions.

### 2.2. Lab and Imaging Investigations

**Figure 2:**
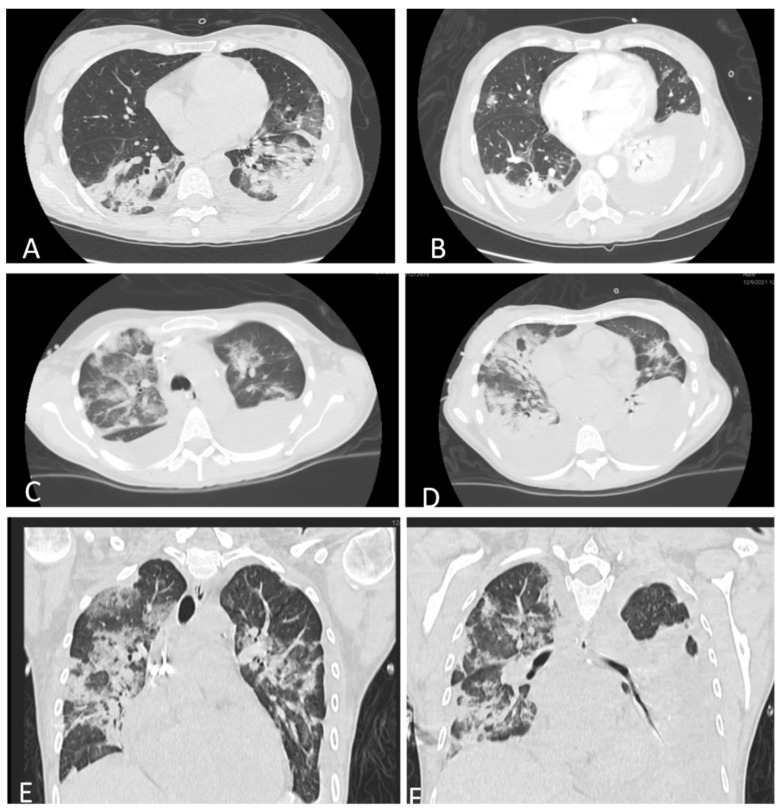
Thoracic CT scan. (**A**) Native thoracic scan showing bilateral COVID-19 pneumonia, affecting 30% of the lungs, and minimal right pleural effusion. (**B**) Contrast-enhanced thoracic CT scan revealing medium right pleural effusion and bilateral COVID-19 pneumonia. (**C**,**D**) Native thoracic CT scan, transversal view with bilateral pleural effusions and bacterial pneumonia. (**E**,**F**) Native thoracic CT scan, sagittal view with large right pleural effusion, inferior right lobe atelectasis, and bacterial pneumonia.

**Figure 3:**
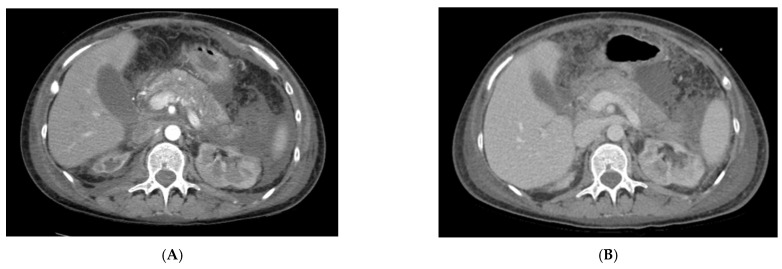
Abdominal CT scan in evolution. (**A**) Initial contrast-enhanced CT scan showing diffuse pancreatic edema and peripancreatic liquid collection. (**B**) Native CT scan revealing inhomogeneous pancreas characteristic of necrotizing pancreatitis, peripancreatic and retroperitoneal collection, and moderate ascites.

On admission, lab tests revealed mild anemia, lymphopenia, and inflammatory syndrome defined by increased levels of C-reactive protein (CRP), D-dimers, and lactate dehydrogenase (LDH). Renal and liver function tests were in the normal range. Extended biological parameters on admission and the trends are listed in Table 1. A thoracic native computer tomography (CT) scan revealed moderate pneumonia affecting 30% of the lungs (Figure 2A). The thoracic CT scan showed bilateral pleural effusions (Figure 2B). Based on these findings, together with the positive RT-PCR and respiratory failure, the diagnosis of a severe form of COVID-19 pneumonia was established. Following delivery, further investigations revealed elevated lipase levels of 2273 IU/L, which were three times higher than the normal value. Clinical and biological data were in favor of acute pancreatitis as indicated by the Atlanta definition [6]. The diagnosis was confirmed by a contrast-enhanced abdominal CT scan, which revealed a pancreas with increased volume and edema, with exudative changes causing liquid collections anterior and posterior to the pancreatic head and within the peritoneal cavity without signs of infection or abscesses (Figure 3A). The diagnosis of AP was established with the following severity scores: SAPS = 17 (2.6%), APACHE = 7 (7.6%), and SOFA = 1 (<10%).

A thoraco-abdominal CT scan repeated on day 10 revealed massive bilateral pleural effusion (Figure 2C–E) and progressing edematous pancreatitis. Due to a high suspicion of infection, cultures were obtained. The results of the cultures are listed in Table 2.

An abdominal US performed on day 16 and day 18 concluded that the patient developed necrotizing pancreatitis complicated by a pancreatic abscess. A CT scan confirmed the presence of the abscess (Figure 3B).

On day 25, slow unfavorable progression indicated the need for a repeat abdominal CT scan, which showed progressive, necrotizing pancreatitis accompanied by intra-abdominal and parietal wall abscesses. The lab test results indicated marked inflammatory syndrome, severe anemia, prolonged coagulation screening, and deteriorating renal function.

**Figure 4:**
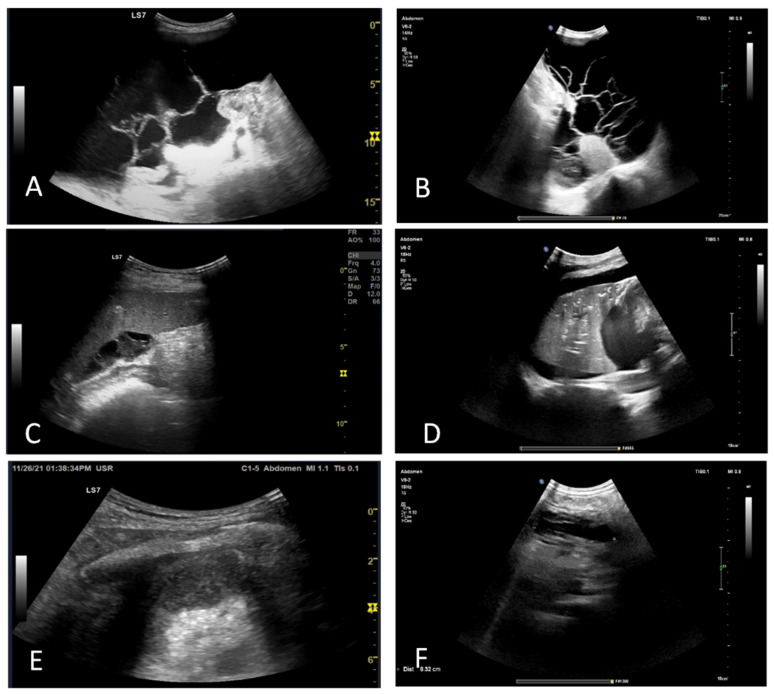
Abdominal and thoracic ultrasound in evolution. (**A**,**B**): Peritoneal effusion with septa. (**C**):Perisplenic cloasonated peritoneal effusion. (**D**): Pleural effusion of the inferior lung lobe, with air bronchograms. (**E**,**F**): Left subphrenic effusion, showing deep enhancement, suggestive of infection (abscess).

On day 30, arterial blood gas analysis showed severe ARDS with an arterial oxygen pressure-to-inspired fraction of oxygen (PaO_2_/FiO_2_) ratio of 50 and mixed respiratory and metabolic acidosis. Increasing inflammatory syndrome was accompanied by worsened renal function. A full blood count revealed severe anemia and thrombocytopenia. An abdominal US showed moderate ascites, retroperitoneal effusion, and necrotizing pancreatitis (Figure 4B). Transthoracic echocardiography and lung US were notable for mild tricuspid and severe mitral regurgitation, mild pulmonary hypertension, an ejection fraction within normal limits, large pleural effusions, and atelectasis (Figure 4C–E). Diagnostic paracentesis was performed for cultures (Table 2). Following the convulsive episode, a head CT scan was performed, and areas of recent ischemia were discovered in the right postero-superior frontal lobe, postero-superior parietal lobe bilaterally, and the right posterior occipital lobe. Heart US was performed, which ruled out endocarditis.

### 2.3. Therapy

Following admission, remdesivir and dexamethasone treatment was initiated as per the National Guidelines for the Treatment of COVID-19. Dexamethasone was also administered for fetal lung maturation. The case was managed by a multidisciplinary team, which included an ICU specialist, internal medicine specialist, gastroenterologist, infectious disease specialist, cardiologist, and general surgeon.

The patient was admitted to ICU, and after consulting with the general surgeon, a conservative approach was chosen. Empiric antibiotic therapy (ABT) was initiated with meropenem and linezolid, as per the specialist consultant, with prior infection screening (cultures). ABT was administered for the treatment of complicated, infected pancreatitis. Empiric ABT was adapted based on clinical progression. Ceftazidime and avibactam therapy was initiated on day 16 for persistent erythema with inflammatory syndrome and leukocytosis. Metronidazole was also initiated, and meropenem and linezolid treatment was stopped. Subsequent ABT was added for sepsis, and the therapy was based on the results of positive cultures. Detailed culture results and ABT can be seen in Table 2.

On day 26, the decision was made to adopt an invasive approach to drain the abscesses in the abdominal cavity and wall. Secondary organ failure was addressed immediately. Packed red blood cells and blood products were transfused to correct anemia and coagulation disorders. Percutaneous US-guided needle aspiration was attempted, with a failure to extract material due to high viscosity and density. As a result, surgical drainage with intraoperative US assistance was performed. Drains were left in situ of the existing cavities to favor drainage from more profound, inaccessible collections. After surgery, the patient was extubated, and due to a marked systemic inflammatory response, which followed surgery and was accompanied by renal failure, continuous renal replacement therapy (CRRT) with hemodiafiltration was performed for 72 h.

Despite surgical and medical treatment, on day 32, noninvasive mechanical ventilation was administered, followed by endotracheal intubation and invasive mechanical ventilation. The pleural effusion was drained and ascites taped for examination. CRRT was again performed for the inflammatory syndrome and renal failure. Subsequent episodes of renal replacement therapy were administered as required based on clinical and biological indicators. Blood transfusions were administered. Subsequent surgical interventions were also required for the drainage and lavage of collections and abscesses. Positive cultures were treated accordingly. After multiple chest drainages and repeated failed attempts to extubate with reintubation, tracheostomy was performed on day 59.

Acute seizure was controlled with first-line benzodiazepine lorazepam 4 mg iv, followed by treatment with valproate 1000 mg and levetiracetam 500 mg per day.

Throughout the entire ICU stay, pressure ulcer prophylaxis, stress ulcer prophylaxis, deep venous thrombosis prophylaxis, and standard skin and oral hygiene were performed. Following stabilization, early enteral nutrition was initiated to maintain enterocyte viability. The control of proteinuria was attempted with angiotensin-converting enzyme (ACE) inhibitors; however, due to acute kidney injury (AKI), it was stopped to regain function. Hypoalbuminemia was addressed by administering continuous infusions of human 20% albumin to maintain oncotic pressure. To address hypoproteinemia, enteral and parenteral nutrition was administered. Energy requirements and nutritional components were adapted. Parenteral nutrition was administered for a short period of time when enteral nutrition was not possible or insufficient. Respiratory physiotherapy and passive and active whole-body physiotherapy were performed. A summary of drugs administered during the ICU stay is presented in Table 3.

### 2.4. Outcome and Follow-Up

Around day 60, following specific and supportive treatment, urine output was regained and was followed by the resolution of generalized edema. Respiratory kinetics and function were also improved, secondary to decreasing pleural effusion as indicated by the presence of bilateral breath sounds. Hemodynamic parameters stabilized, and her baseline tendency to hypertension reoccurred. While the distention persisted, palpation revealed a soft and nontender abdomen. Tolerance of enteral nutrition was regained. On day 71, the patient experienced an episode of generalized tonic and clonic convulsions accompanied by respiratory and hemodynamic impact. After regaining consciousness, no neurological deficit was recorded. Following consistent positive progression, the patient was discharged from ICU on day 78 with persistent but much diminished requirements for oxygen therapy, controlled hypertension, efficient UO, and good tolerance to enteral nutrition. A right gluteal pressure ulcer was present on discharge. The patient was discharged after 3 months of hospitalization. At discharge, she tested negative for SARS-CoV-2 infection and showed no signs of further infection.

## 3. Discussion

This case illustrates a severe complication of SARS-CoV-2 infection in a pregnant nonvaccinated patient with cardiovascular and renal comorbidities. In our case, the sudden onset of pain was associated with increased levels of amylase and lipase. As the patient was postpartum, the CT scans were initially used to identify other possible causes of abdominal pain after surgical intervention and, during hospitalization in ICU, to characterize the complications (necrosis and abscesses). In the early phase, during the first week of evolution, the systemic inflammatory response (SIRS) is in the foreground, while the late phase (the 2nd week) is dominated by persistent organ failure and local complications [6].

Viral pancreatitis is a diagnosis of exclusion, meaning that other possible etiologies must first be ruled out. In our case, gallstones, alcohol consumption, hypertriglyceridemia, and hypercalcemia were easily ruled out by CT scans and lab testing. In pregnancy, the most frequent causes of AP are similar to those in the general population, that is, gallstones, alcohol abuse, and hereditary, and the causes of almost 20% of cases remain unclear [12]. Cases of viral SAP are described in the literature, but always as a diagnosis of exclusion. In our case, SARS-CoV-2 infection was implicated. Unfortunately, the variant was not determined, but the case was diagnosed during the Delta variant wave (November 2021); as such, we can only hypothesize that, based on the severity of the symptoms and the epidemiologic distribution, the Delta variant was responsible for the disease. However, the diagnosis of drug-induced pancreatitis is not quite straightforward, and it is classified based on the number of cases reported, demonstration of a consistent latency period, and possible reaction when the drug is reintroduced [13]. However, the administration of a certain drug may not always be blunt, even in suspected cases. AP may develop within a few weeks after beginning a drug associated with an immunologically mediated adverse reaction; in this setting, the patient may also have a rash and eosinophilia. In contrast, other drugs could cause AP development only after many months of use as a result of the chronic accumulation of toxic metabolic products [14]. Most of the published evidence is based on case reports, and, hence, it is very important to rule out all other causes before attributing a drug as the cause of acute pancreatitis [15]. In our case, the chronic medication (antiaggregant and antihypertensive medication) was introduced years before presentation and cannot be, at least theoretically, implicated as the cause of the AP. For example, our patient was on α-methyldopa for the management of hypertension before becoming pregnant. There are four cases that associate α-methyldopa with AP, with the onset symptoms in less than 2 weeks after initiation of the therapy and a mild outcome [13]. The drugs that could be implicated in AP are dexamethasone, remdesivir, and ABT. There is only one case described in literature that links the administration of remdesivir to AP, and it was recently published by Khadka S [16]. In that case, the clinical onset of pain began on the 4th day of remdesivir administration and disappeared within 3 days after withdrawal of remdesivir, a characteristic aspect of the evolution of AP [16]. As for dexamethasone, it was shown to offer protection against AP via the upregulation of pancreatitis-associated proteins, thus decreasing AP severity [17]. Before the onset of AP, our patient was given ABT to prevent bacterial infection after cesarean section. The changing of ABT did not influence the development of AP. Even though drug-induced AP is hard to exclude, in our case, the onset and evolution of AP were not linked to any medication, and the severity was not typical for this kind of AP.

Enteral nutritional (EN) support therapy has been shown to improve clinical outcomes in patients with SAP in numerous studies, and it has become a major component of standardized therapy [18]. EN support has been widely employed as the primary choice of nutritional support due to its ability to protect the functioning of the intestinal mucosal barrier through a variety of ways. According to the newest American Nutrition Society guidelines [19], EN support can begin soon after active fluid resuscitation (ebb phase), typically after 24–48 h. Enteral tube feeding is possible. Our patient began minimal enteral nutrition after stabilization with progressively increasing doses and a rate adapted to her tolerance and requirements. During the periods of full compliance, the patient received balanced oral fluid nutrition containing protein, carbohydrates, vegetable oils, minerals, vitamins, and trace elements. Enteral nutrition was also administered via a feeding tube during the period when she was intubated. Due to increased intra-abdominal pressure and gastrointestinal dysfunction, the beginning and maintenance of EN is challenging in SAP patients. In SAP patients, an objective examination of gastrointestinal function aids in determining the most effective EN support. Unfortunately, no objective signs to measure gastrointestinal function in SAP patients is available as stipulated in the International Guidelines for Intensive Care Medicine [20] and Gastroenterology [21].

In the early phase, during the first week of evolution, the systemic inflammatory response (SIRS) is in the foreground, while the late phase (the 2nd week) is dominated by persistent organ failure (MODS) and by local complications [6]. SIRS and MODS may occur in the early stages of SAP due to the enormous release of inflammatory mediators. As a result, the focus of early SAP treatment in the ICU should be on organ function support [22]. To avoid puncture-related problems, including subsequent infection and bleeding, surgical treatment for asymptomatic local issues, such as acute peripancreatic fluid collection and acute necrotic collection, is not indicated [22]. In symptomatic patients (e.g., gastrointestinal compression-related symptoms with decreased EN support or feeding) or in patients with secondary infection, ultrasound or CT-guided biopsy can be employed. Prokinetic drugs, such as IV metoclopramide and IV erythromycin, can be administered early to patients with high intra-abdominal pressure to stimulate gastrointestinal motility or achieve gastrointestinal decompression. In the case of progressively rising intra-abdominal pressure or nonresponsive abdominal compartment syndrome, laparotomic decompression might be performed with caution and a multidisciplinary approach. The postsurgical incision should be temporarily covered with a patch or other materials to avoid complications such as intestinal injury [23]. Initially, our patient was treated conservatively, and she was constantly monitored to identify the emergence of co-infection.

In the late phase, if sepsis is caused by pancreatic infection, the mortality rate could rise up to 80%. If left untreated, infectious pancreatic necrosis with MODS has a patient fatality rate of up to 100% [24]. As mentioned before, throughout our patient’s conservative treatment, the risk of infection was continuously monitored. The signs of infection were indicated on day 16 after hospitalization by fever, high blood pressure, intra-abdominal pressure, leukocytosis, elevated procalcitonine, and positive cultures. In this scenario, fine-needle aspiration was not possible, and repeated blood cultures and CT scans with IV contrast were performed. The surgical intervention was performed on the 25th day of hospitalization.

Thrombotic events (TEs) are a serious issue for hospitalized patients due to increased risks of deep venous thrombosis (DVT) and secondary pulmonary embolism (PE), carrying a 12% higher fatality rate [25]. Our patient presented many factors precipitating a hypercoagulable state. Firstly, she suffered from an undiagnosed nephropathy with documented proteinuria, which was above 3.5 g/day during her hospital stay. Nephrotic syndromes are not only associated with DVT but may also lead to stroke [26]. Secondly, our patient was known to have minor congenital thrombophilia. The third factor for hypercoagulability was COVID-19. Vascular endothelial damage, immobility caused by critical illness, and severe inflammation account for the hypercoagulable state. The witnesses of the hypercoagulability state in this case are the elevated D-dimer levels. To reduce the risk, prophylactic anticoagulation is warranted; however, this increases the risk of bleeding, especially when associated with aspirin or when renal impairment is present [27,28]. Rather than pharmacologic prophylaxis, mechanical intermittent compression stocking and early mobilization were adopted in our case.

Inflammation is present in both COVID-19 and AP. Ferritin is a biological marker that is associated with mortality and is used as a prognostic tool in patients with COVID-19 [29]. Elevated levels of ferritin, termed hyperferritinemia, is defined by levels above 500 ng/mL. On admission, very high levels such as >3000 ng/mL are associated with 30 day mortality. Furthermore, trends also prove valuable, with decreasing values signaling recovery [30]. Reports on the association of COVID-19-induced hyperferritinemia and secondary AP are scarce. The mechanism by which ferritin is increased during inflammation relies on the protein hepcidin, which affects iron metabolism by blocking the only iron exporter available, ferroportin [31]. Iron overload, with saturated deposits, triggers ferroptosis, a form of cell death. Furthermore, the process of cell death is propagated by damage-associated molecular patterns of oxidative stress and stimulates inflammation to maintain the vicious circle [32]. Our patient had mildly elevated levels on admission, which, by day 7, increased to levels above 500 ng/mL (634 ng/mL), peaking on day 25 when surgical treatment was deemed necessary. After control of the disease was gained, a downward trend was seen, although it remained elevated.

### 3.1. Particularities of the Case

The particularities of the case are as follows:

- SAP complicated by a subdiaphragmatic abscess and MODS in a postpartum patient with a severe form of SARS-CoV-2 infection;
- Antepartum cardiovascular comorbidities and thrombophilia that may have favored the severity of SARS-CoV-2 infection and the development of SAP;
- Multiple in-hospital infections at different sites that eventually responded to ATB;
- Favorable development despite the worse prognosis.

### 3.2. Clinical Implications with Recommendations for Clinicians

The clinician should be aware of the association of SARS-CoV-2 and acute pancreatitis in pregnant women. We highlight the importance of abdominal pain evaluation in postpartum patients with COVID-19, as AP could be the underlying cause of the pain. The clinical presentation and the presence of inflammation could be misleading, and, therefore, lab (lipase) and imaging investigations (abdominal ultrasound and CT scans) play a key role in the diagnosis, as recommended by the international guidelines. Lipase and amylase should be included in laboratory routine tests to evaluate whether there is a need for abdominal imaging in pregnant or postpartum patients with COVID-19.

A positive diagnosis is not straightforward, as biliary calculus, alcohol consumption, and drug toxicity must be excluded. The evaluation of severe prognosis using severity scores is recommended during the first 48 h from the onset of pain. Once the diagnosis of SAP is established, patients should be carefully monitored in ICU for SIRS, MODS, and thrombotic and infectious complications. Periodic imaging evaluation (daily ultrasound monitoring and abdominal CT scan when the clinical and biological variables demand it) is useful to identify the local complications. A close collaboration with a gastroenterologist for minimal invasive procedures is advised, as surgical interventions require general anesthesia, which bears a greater risk of mortality in COVID-19 patients than in the general population. Imaging evaluation allows the physician to establish the precise moment for surgical intervention. Parenteral nutritional support is required if oral intake is not possible within 1 week, with the close surveillance of lipid and triglyceride levels. Enteral nutrition must be commenced as soon as possible to maintain enterocyte viability to prevent bacterial translocation. ABT should begin as soon as an infection is suspected, while measures are taken to identify the cause of the infection. Empirical ATB without any proven infection is not recommended. Prophylactic or therapeutic anticoagulation should be administered with caution, carefully monitoring the bleeding risk (hemoglobin, hemodynamic surveillance status for transfusion in accordance with patient’s blood management protocol).

### 3.3. Literature Review

The literature data regarding SAP during COVID-19 infection and pregnancy are scarce. A search on PubMed using the keywords “SARS-CoV2”, “COVID-19”, “pregnancy”, and “acute pancreatitis” from 2020 to 2022 identified seven results, among which only three publications fulfilled all the criteria. These publications are case reports of pregnant patients without known comorbidities affected by COVID-19. Thus, this is the first case of complicated SAP in a pregnant woman with moderate/severe SARS-CoV-2 infection and comorbidities, who had a good outcome. One of the case reports of AP in pregnancy occurred in a patient with diabetes, and amylase and lipase levels did not reach three times the upper limit of the normal range, which is the threshold recommended by the guidelines for a positive diagnosis of AP [33]. The other was published by Narang K et al. and described a mild form of interstitial edematous AP that improved significantly immediately postpartum [34]. Handaya Y et al. referred to a case of mild AP caused by migratory gallstones and acute cholecystitis in a 37-year-old pregnant patient with a good outcome [35]. The details of the presented cases are summarized in Table 4.

## 4. Conclusions

We presented the first case of complicated SAP in a pregnant woman with moderate/severe SARS-CoV-2 infection and cardiovascular and renal comorbidities, who had a good outcome. Abdominal pain in the postpartum period could be misleading, and inflammation was triggered by COVID-19; therefore, the diagnosis was based on lab testing and imaging. SAP was complicated by pancreatic abscesses treated by both ABT and surgery. During hospitalization, the patient presented multiple infections favorized by the immunosuppressed postpartum and COVID-19 state. This report, along with the short literature review, should make clinicians aware of the association of SARS-CoV-2 and acute pancreatitis in pregnant women.

## Figures and Tables

**Table 1:** Serological parameters and values over time, from admission to day 60.

Lab Parameters	Admission (Day 1)	Dx of PA (Day 7)	Necrosis (Day 16)	Surgery (Day 25)	Post-Op. (Day 30)	Post-Op. (Day 60)	Lab Range
WBC × 10^9^/L	15.00	21.97	19.96	9.16	9.23	7.72	4–10
Ly × 10^9^/L	1.32	0.68	0.68	1.89	1.12	2.0	1.1–3.5
Platelets × 10^9^/L	-	281	154	219	241	244	150–400
CRP (mg/L)	1.184	3.287	3.174	2.36	1.046	0.77	<0.05
PCT (ng/mL)	0.457	0.504	15.647	0.112	0.856	0.94	<0.06
D-dimers (ng/mL)	1034	1284	1551	-	682	1039	<243
Fbg (mg/dL)	722	439	596	367	211	321	200–400
Hs-TnI (pg/mL)	14.7	19.4	20.7	-	20.7	-	<11.6
Ferritin (ng/mL)	153	634	824	1625	1240	1811	10–120
Hb (g/dl)	10.3	9.9	8.6	9.3	8	8	12–15.5
Amylase (U/L)	2407	384	83	108	44	120	22–80
Lipase (U/L)	2773	378	58	85	56	80	<39
GGT (U/L)	51	15	100	53	34	44	<38
ALT (U/L)	18	6	4	37	6	84	<35
Direct Bilirubin (mg/dL)	0.13	0.1	0.31	0.2	0.19	0.12	<0.2
Total Bilirubin (mg/dL)	0.37	0.35	0.86	0.81	0.85	0.54	0.3–1.2
TGL (mg/dl)	-	179	243	-	280	-	<150
LDH (U/L)	364	643	261	197	316	-	<247
PaO_2_ (mmHg)	-	113	98.5	-	65	-	80–100
PaO_2_/FiO_2_	-	376	328	-	72	-	>300
Ca (mmol/L)	1.86	2.1	2.2	-	2.315	-	2.2–2.65
Creatinine (mg/dL)	1.79	1.70	1.93	3.47	2.34	3.15	0.51–0.95
Alb (g/dL)	2.49	3.02	3.29	3.86	3.74	3.7	3.5–5.2
Proteinuria/24 h	-	6572 mg	-	-	9000 mg	-	-

Alb: albumin; ALT: alanine aminotransferase; CRP: C-reactive protein; Fbg: fibrinogen; GGT: gamma glutamyl transpeptidase; Hb: hemoglobin; Hs-TnI: high-sensitivity troponin I; LDH: lactate dehydrogenase; Ly: lymphocytes; PaO_2_: partial pressure of oxygen; PaO_2_/FiO_2_: arterial oxygen partial pressure-to-inspired fraction of oxygen ratio; PCL: procalcitonin; TGL: triglyceride; WBC: white blood cells; Post-op: postoperative; -: absent data.

**Table 2:** Bacteria and fungus growth from cultures, including antibiograms.

Days of Hospitalization	Culture Type and Germs	Antibiogram
16.11 (day 9)	Urinary cultures: *Enterococcus faecium* VRE	Sensitive to linezolid and tigecycline
23.11 (day 16)	Hemoculture: *Klebsiella pneumoniae* spp.	Sensitive to colistin
2.12 (day 25)	Uterus secretion: *Escherichia coli*	Sensitive to meropenem and colistin
11.12 (day 34)	Urinary cultures: *Candida* spp.	Sensitive to fluconazole
19.12 (day 42)	Urinary cultures: *Enterococcus faecium* VRE	Sensitive to linezolid and tigecycline
19.12 (day 42)	Tracheobronchial aspirate: *Acinetobacter baumannii*	Sensitive to colistin and tigecycline
11.01 (day 65)	Pleural liquid: *Staphylococcus* *aureus* MSSA, MLSBc	Resistant to amoxicillin/clavulanate and gentamicin

MSSA: methicillin-susceptible staphylococcus; MLSBc: macrolide–lincosamide–streptogramin B chromosomal resistance; spp.: species; VRE: vancomycin resistant.

**Table 3:** Summary of drug therapy during hospitalization.

Drug	Dose and Route of Administration	Duration of the Therapy (Days)
Remdesivir	100 mg iv	5 days
Dexamethasone	16 mg iv	7 days
Aspirin	100 mg po	10 days
Clexane	0.4 mL sc	During the entire hospitalization
Meropenem	1000 mg iv	From the 11th day for 10 days
Linezolid	600 mg iv	From the 11th day for 10 days
Zavicefta (ceftazidime and avibactam sodic)	2/0.5 g iv 3×/day	From the 16th to day 26th
Colistin	4.5 mil IU 2×/day	Started on day 48
Metronidazole	500 mg iv 3×/day	From the 11th day for 10 days
Lorazepam	4 mg iv	1 day
Valproate	1000 mg	10 days from day 32
Levetiracetam	500 mg per day.	10 days from day 32
Albumin 20%	240 mL/day	If albumin levels were <3 g/dL

**Table 4:** Articles related to COVID-19, AP, and pregnancy.

Authors/Journal/Year	Pregnancy Age	PA Severity	PA Etiology	Outcome
1. Narang K et al., *Obstet Gynecol.* 2021	21-year-old primigravid patient at 33 weeks of gestation	Mild edematous pancreatitis	Viral	Good
2. Rabice SR. et al., *Case Reports in Women’s Health* 2020	36-year-old primigravid patient at 33 weeks of gestation	Mild edematous pancreatitis	Viral	Good
3. Handaya Y. et al., *Ann Med Surg (Lond)* 2021	37-year-old primigravid patient at 22 weeks of gestation	Mild edematous pancreatitis	Biliary (gallstones and acute cholecystitis)	Good

## Data Availability

Not applicable.
